# A Pancreatic Hydatid Cysts Causing Recurrent Acute Pancreatitis Mimicking a Pancreatic Pseudocyst: A Case Report

**DOI:** 10.7759/cureus.36402

**Published:** 2023-03-20

**Authors:** Vasistha Jajal, Hirdaya Nag, Sugumaran K

**Affiliations:** 1 Surgical Gastroenterology, Govind Ballabh (GB) Pant Institute of Postgraduate Medical Education and Research, New Delhi, IND

**Keywords:** laparoscopic partial pericystectomy, cystic lesions of pancreas, pancreatic pseudocyst, recurrent acute pancreatitis, hydatid cyst of pancreas, hydatid disease

## Abstract

Hydatid disease is a zoonotic disease, mainly prevalent in endemic countries. The liver and lungs are the most commonly affected organs in hydatid disease. Primary hydatid cyst of the pancreas is rare, and pancreatitis due to hydatid cyst has rarely been listed in the literature. Hydatid cyst of the pancreas is difficult to diagnose preoperatively. It can be misdiagnosed as a pseudocyst of the pancreas. We report the case of a 32-year-old female patient who presented with recurrent acute pancreatitis. Following preoperative imaging, the primary impression was a pancreatic pseudocyst. On further evaluation with endoscopic ultrasound-guided fine needle aspiration (FNA) and hydatid serology, she was diagnosed with a pancreatic hydatid cyst. Laparoscopic partial pericystectomy was performed. In literature, pancreatic hydatid cysts were mainly treated with a traditional open surgical approach. A minimally invasive surgical approach is evolving as an option in selected cases of pancreatic hydatid cysts.

## Introduction

Echinococcosis or hydatid disease is a zoonotic parasitic disease caused by the tapeworm Echinococcus [1]. Dogs are the definitive hosts, while livestock is the intermediate host. Humans are infected as an accidental intermediate host after the inadvertent ingestion of Echinococcus eggs in canine feces. Hydatid disease has a global distribution, with a worldwide annual incidence rate of 1-200 per 100,000 population [2]. It is endemic in many sheep- and cattle-raising geographic areas and continues to be a major public health issue where agriculture and stockbreeding are primary sources of income, including Mediterranean countries, the Middle East, Eastern Europe, and South America. The prevalence of hydatid disease in endemic areas is 1% to 10%; however, the incidence rate in hospitals is about 1,000 times less than the prevalence as only a small proportion of symptomatic patients seek medical care [3].

The liver (70%) is the most commonly affected organ in hydatid disease, followed by the lungs (20%) [4]. Pancreatic hydatid cysts are rare accounting for 0.2% of the cases of overall hydatid disease [5]. Pancreatic hydatid cysts may develop as primary (involving the pancreas only) or secondary (with multiple organ involvement) diseases [6]. Acute pancreatitis secondary to hydatid parasitosis is reported in less than 2% of the cases of pancreatic hydatid cysts in endemic countries [7]. Only a few cases of pancreatitis secondary to pancreatic hydatid disease were reported in the literature [1].

Despite the advanced radiological imaging, preoperative diagnosis of pancreatic hydatid cysts is challenging and may be mistaken as a cystic tumor or pseudocyst of the pancreas. Surgery, conservative or radical, is still the treatment of choice for hydatid disease in all locations [8].

Here, we report a case of pancreatic hydatid cyst causing recurrent acute pancreatitis, which was treated by laparoscopic partial pericystectomy.

## Case presentation

**Figure 1:**
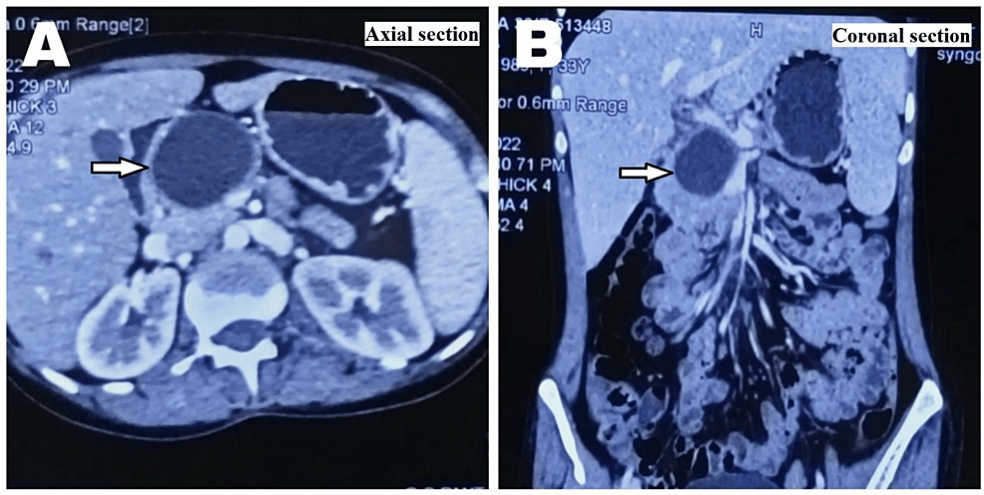
Contrast-enhanced CT abdomen showing peripherally enhancing collection in the head of the pancreas likely a pseudocyst: (A) axial section and (B) coronal section. CT, computed tomography

A 32-year-old female patient presented with a chief complaint of upper abdominal pain, moderate in intensity for six months. She had an associated history of nausea, occasional vomiting episodes, and weight loss (4-5 kg in the last six months). She had no history of fever, jaundice, or abdominal distension. The intensity and frequency of pain gradually increased over time, requiring injectable analgesics. She had no history of contact with dogs or livestock such as cattle, sheep, and goats. On general physical examination, she had no signs of pallor or icterus. Her vital signs were stable. Per abdominal examination, the abdomen was soft, with mild epigastric tenderness. Approximately 7 cm × 6 cm-sized, ill-defined, firm, mildly tender, immobile mass was palpable in the epigastric region, with no other abnormal findings. On laboratory examination, her hemoglobin level was 9.9 g/dL (reference value 12-15 g/dL) and her total leucocyte count was 7,800 mm^-3 ^(reference value 5,000-10,000 mm^-3^), with a normal absolute eosinophil count. Her liver and renal function tests were within normal limits. Ultrasound abdomen revealed a 4.0 cm × 5.0 cm well-defined anechoic cystic lesion noted adjacent to the head of the pancreas, suggestive of a pseudocyst of the pancreas. She was further evaluated with contrast-enhanced CT (CECT) abdomen and magnetic resonance cholangiopancreatography (MRCP). CECT abdomen revealed a peripherally enhancing collection seen in the head of the pancreas measuring 4.1 cm × 4.5 cm × 4.3 cm in size likely pseudocyst (Figure 1). The pancreatic body and tail appeared atrophic and showed dilated main pancreatic duct (MPD) measuring 5.8 mm in caliber.

**Figure 2:**
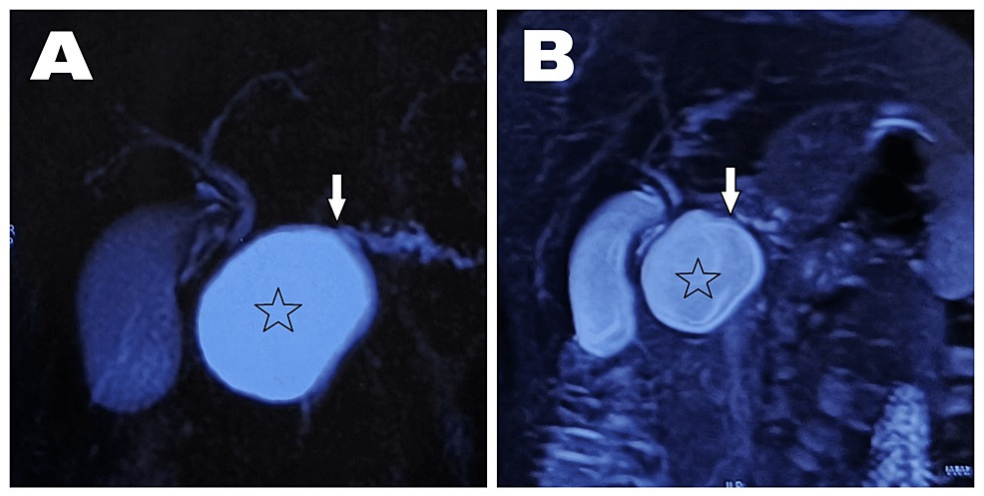
(A and B) MRCP images revealing a well-defined cystic lesion (star) occupying the head of the pancreas, along with dilated MPD with no communication between the cystic lesion and MPD (arrow). MRCP, magnetic resonance cholangiopancreatography; MPD, main pancreatic duct

MRCP revealed a fluid-filled, well-defined capsulated cystic lesion measuring 46 mm × 44 mm × 40 mm, occupying the pancreatic head region with dilated MPD measuring 6 mm in diameter (Figure 2). No communication was appreciated between the cystic lesion and MPD. Findings were suggestive of a cystic lesion of the pancreas, likely a pseudocyst of the pancreas. Upper GI endoscopy revealed an extrinsic impression over the medial wall of the second part of the duodenum, with remaining normal findings. Serum amylase and lipase levels were 191 U/L (reference value 25-125 U/L) and 64 U/L (reference value 13-50 U/L), respectively. Thus, she was diagnosed with a cystic lesion in the head of the pancreas, causing recurrent acute pancreatitis, likely a pseudocyst of the pancreas.

**Figure 3:**
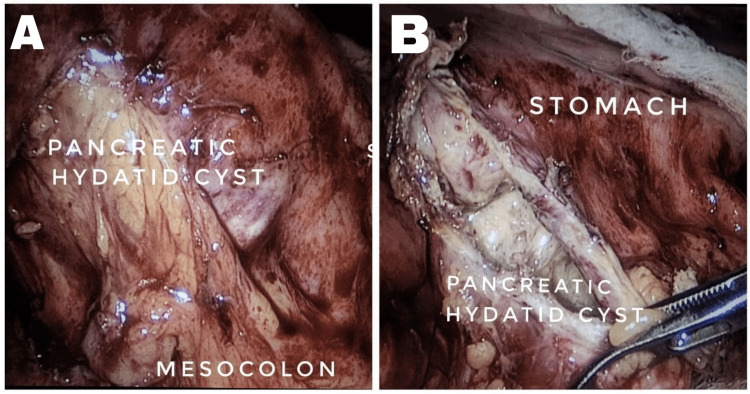
Intraoperative images: (A) an approximately 5 cm × 5 cm-sized cystic lesion in the head and proximal body of pancreas; (B) opened hydatid cyst cavity, germinal layer seen, and no evidence of daughter cysts.

She was further evaluated with endoscopic ultrasound-guided fine needle aspiration (FNA) from a pancreatic cystic lesion to confirm the diagnosis, which revealed numerous fragments of parasitic scolices and hooklets, suggestive of hydatid cysts. The aspirated fluid amylase level was normal. As the FNA report was suggestive of a hydatid cyst, hydatid serology was done and reported as 89.3 U/mL (normal value <15 U/mL). Thus, she was diagnosed with a pancreatic hydatid cyst. She underwent laparoscopic surgery for the primary pancreatic hydatid cyst. Intraoperatively, an approximately 5 cm × 5 cm × 6 cm-sized cystic lesion is present in the head and proximal body of the pancreas. Cyst anterosuperior abutting greater curvature of the stomach with loss of fat planes is shown in Figure 3. On aspiration, it was clear fluid. On opening, an endocyst was identified, with no evidence of a daughter cyst. The cyst cavity was irrigated with hypertonic saline and 10% betadine solution with a contact period of 10 minutes. Laparoscopic partial pericystectomy was performed. No communication with the MPD was identified. The cyst wall was sent for histopathological examination and was confirmed to be a hydatid cyst.

The postoperative course remained uneventful. She was allowed a liquid diet on postop day 2 and a soft diet on postop day 3 and was discharged on postop day 4. Albendazole (400 mg twice a day) was prescribed for three months. She was readmitted two weeks after discharge with upper abdominal pain and fever. Per the abdomen CT, there was mild tenderness in the epigastric region. Repeat investigations showed a total leucocyte count of 21,200 mm^-3^. The serum amylase level was normal. Other routine blood investigations were within normal limits. CECT of the abdomen was performed, which revealed a well-defined collection with air foci within, in relation to the head of the pancreas. She was managed expectantly with broad-spectrum antibiotics, and she gradually improved and was discharged. Albendazole was continued. She was well after six months of follow-up with no recurrence.

## Discussion

Hydatid cysts can theoretically develop in any organ and structure of the body [1]. Primary pancreatic hydatid disease is rare (accounting for <1% of all hydatid diseases). It may develop through hematogenous dissemination, local spread via pancreaticobiliary ducts, or more rarely through lymphatic spread [9]. A pancreatic hydatid cyst is solitary in 90% of the cases, out of which 50% to 57% occur in the head, 24% to 34% in the body, and 16% to 19% in the tail of the pancreas [7]. The pathogenesis of pancreatitis in pancreatic hydatid remains unclear. Two hypotheses are present: (1) compression of the main pancreatic duct by the cyst itself [10] and (2O obstruction of the main pancreatic duct by scolices migrated from the cyst [11].

Clinical presentation of pancreatic hydatid varies according to size, anatomical location, and potential complications. Diagnosis of pancreatic hydatid is based on imaging as there are no specific symptoms or signs of pancreatic hydatid cysts. In imaging, due to nonspecific radiological features, the diagnosis of pancreatic hydatid cysts is very difficult, and it can be misdiagnosed unless hydatid disease is suspected. Clinical suspicion along with imaging and serological test increases the diagnostic yield [12].

Surgery, along with a perioperative anthelmintic agent, is the treatment of choice for hydatid disease in any location. Surgical treatment is indicated in symptomatic hydatid disease and patients with active or complicated hydatid disease. For pancreatic hydatid, depending upon the cyst location and communication with the main pancreatic duct, several surgical procedures have been suggested, ranging from cyst fenestration or partial pericystectomy to Whipple’s pancreaticoduodenectomy or distal pancreatectomy in the case of cyst communication with the main pancreatic duct [7]. Medical treatment in the perioperative period reduces the risk of recurrence [13]. Albendazole is the most effective anthelmintic agent. Although there are many publications in the literature about the laparoscopic approach in hydatid cysts of the liver, there are only a few reports addressing this approach in pancreatic hydatid disease [8].

## Conclusions

Hydatid disease of the pancreas though rare pathology may be a causative factor for recurrent acute pancreatitis and should be considered as a differential diagnosis in case of cystic lesions of the pancreas in endemic countries. Hydatid cysts may easily be confused with the pseudocyst of the pancreas. Although the main surgical approach is still traditional open laparotomy, the laparoscopic approach can be considered in selected cases of pancreatic hydatid cysts.
